# Astaxanthin decreased oxidative stress and inflammation and enhanced immune response in humans

**DOI:** 10.1186/1743-7075-7-18

**Published:** 2010-03-05

**Authors:** Jean Soon Park, Jong Hee Chyun, Yoo Kyung Kim, Larry L Line, Boon P Chew

**Affiliations:** 1 School of Food Science, Washington State University, Pullman, WA 99164-6376 USA; 2 Food and Nutrition, Inha University, Incheon, Korea; 3 La Haye Labs, Inc, Redmond, WA, USA

## Abstract

**Background:**

Astaxanthin modulates immune response, inhibits cancer cell growth, reduces bacterial load and gastric inflammation, and protects against UVA-induced oxidative stress in *in vitro *and rodent models. Similar clinical studies in humans are unavailable. Our objective is to study the action of dietary astaxanthin in modulating immune response, oxidative status and inflammation in young healthy adult female human subjects.

**Methods:**

Participants (averaged 21.5 yr) received 0, 2, or 8 mg astaxanthin (n = 14/diet) daily for 8 wk in a randomized double-blind, placebo-controlled study. Immune response was assessed on wk 0, 4 and 8, and tuberculin test performed on wk 8.

**Results:**

Plasma astaxanthin increased (*P *< 0.01) dose-dependently after 4 or 8 wk of supplementation. Astaxanthin decreased a DNA damage biomarker after 4 wk but did not affect lipid peroxidation. Plasma C-reactive protein concentration was lower (P < 0.05) on wk 8 in subjects given 2 mg astaxanthin. Dietary astaxanthin stimulated mitogen-induced lymphoproliferation, increased natural killer cell cytotoxic activity, and increased total T and B cell subpopulations, but did not influence populations of T_helper_, T_cytotoxic _or natural killer cells. A higher percentage of leukocytes expressed the LFA-1 marker in subjects given 2 mg astaxanthin on wk 8. Subjects fed 2 mg astaxanthin had a higher tuberculin response than unsupplemented subjects. There was no difference in TNF and IL-2 concentrations, but plasma IFN-γ and IL-6 increased on wk 8 in subjects given 8 mg astaxanthin.

**Conclusion:**

Therefore, dietary astaxanthin decreases a DNA damage biomarker and acute phase protein, and enhances immune response in young healthy females.

## Introduction

Studies have reported important functions played by natural carotenoids in regulating immunity and disease etiology [1,2]. Specifically, interest in the biological activity of astaxanthin, an oxycarotenoid found in high amounts in the carapace of crustaceans and in the flesh of salmon and trout, has increased in recent years. In vitro studies have demonstrated that astaxanthin is several fold more active as a free radical antioxidant than β-carotene and α-tocopherol [3].

Using a rodent model, we [4] and others [5,6] have demonstrated that astaxanthin stimulated immune response in mice. Mice supplemented with astaxanthin had increased *ex vivo *splenocyte antibody response to T-dependent antigens [6], lymphoblastogenic response and cytotoxic activity [4]. Moreover, these studies also showed that astaxanthin was consistently more active than other carotenoids such as β-carotene, lutein and canthaxanthin.

In addition to immunoregulatory activity, astaxanthin also inhibited mammary tumor growth. We [7] reported that dietary astaxanthin inhibited mammary tumor growth in mice. Astaxanthin has been shown to reduce bacterial load and gastric inflammation in *Helicobacter pylori*-infected mice [5], and to protect against UVA-induced oxidative stress [8].

Immune cells are particularly sensitive to oxidative stress due to a high percentage of polyunsaturated fatty acids in their plasma membranes, and they generally produce more oxidative products [1]. Overproduction of reactive oxygen and nitrogen species can tip the oxidant:antioxidant balance, resulting in the destruction of cell membranes, proteins and DNA. Therefore, under conditions of increased oxidative stress (e.g. during disease states), dietary antioxidants become critical in maintaining a desirable oxidant:antioxidant balance. While studies on the immunomodulatory role of dietary astaxanthin have been reported in rodents, similar studies in humans are not available. We hypothesize that dietary astaxanthin will act as a potent antioxidative and anti-inflammatory agent; through these and other mechanisms, astaxanthin can enhance immune response. Our objective is to study the possible immune-enhancing, antioxidative and anti-inflammatory activity of dietary astaxanthin in humans.

### Subjects and methods

#### Study participants and study design

Free-living healthy female college students with an average age of 21.5 yr (20.2-22.8 yr) and BMI of 21.6 (16.3-27.5) were participants in this study. Participants were recruited from Inha University (Seoul, Korea) through flyers and emails, and all were native Koreans. Subjects with a history of diabetes, alcohol abuse, cancer or smoking were excluded; exclusion criteria also included those taking antioxidant supplements. Prior to the initiation of dietary supplementation, a three-day dietary record was obtained from each subject who provided informed consent. During the study, subjects were allowed to consume their normal diets but were advised to refrain from eating astaxanthin-rich foods such as salmon, lobster, and shrimp. Subjects were ranked based on BMI (age was within a very narrow range) and groups of 3 participants with similar BNI were randomly assigned to receive daily: 0 (control; Con), 2 mg (2Asta), or 8 mg (8Asta) astaxanthin (109 g astaxanthin complex/kg oleoresin concentrate from *Haematococcus pluvialis*, astaZanthin™, La Haye Laboratories Inc., Redmond, WA) (n = 14 subjects/diet) for 8 wk in a double-blind, placebo-controlled study. Astaxanthin was administered as a softgel capsule taken every morning, and all softgel capsules were externally identical. Blinding was further ensured by assigning consecutive numbers to the dietary treatments and maintaining a master list until the study was completed. The astaxanthin complex used in this study came from a supercritical CO_2 _extract of *Haematococcus pluvialis*. Astaxanthin in the *H. pluvialis *extract is entirelythe 3S, 3S' enantiomer, and is primarily monoesterified with smaller quantities of diester and free astaxanthin. The astaxanthin complex also contains small amounts (<15%) of mixed carotenoids including lutein, β-carotene and canthaxanthin. To minimize subject-to-subject and assay-to-assay variation due to different sampling days, blood was drawn from all 42 subjects on one day for each of wk 0, 4 and 8. Immune function and oxidative status was assessed within 24 h of blood collection. All procedures were approved by the Institutional Review Board (IRB #4421) of Washington State University.

### Analytical procedures

#### HPLC

Astaxanthin content in plasma was analyzed by reverse phase HPLC (Alliance 2690, Waters, Milford, MA) as previously described [9]. Trans-β-apo-8'carotenal (Sigma Chem. Co., St. Louis, MO) was used as the internal standard. Mobile phase used was acetonitrile:methanol:water, 47:47:16 (v/v/v), and samples were eluted through a 5-μm spherical C-18 column (3.9 × 150 mm Resolve, Waters, Milford, MA) at a flow rate of 1.5 mL/min. Absorbance was monitored at 492 nm on a photo diode array detector.

#### Lymphoproliferation

The proliferation response of peripheral blood mononuclear cells to phytohemagglutinin (2 and 10 mg/L final concentration), concanavalin A (2 and 10 mg/L), and pokeweed mitogen (1 and 5 mg/L) was assessed using whole blood cultures (to mimic *in vivo *conditions) as previously described [10]. Results were calculated as stimulation index.

#### Natural killer cell cytotoxic activity

Effector cells (peripheral blood mononuclear cells) and target (K562) cells were cultured at effector:target ratios of 5:1 and 10:1 in DMEM (Sigma, St. Louis, MO) containing 100 mL/L fetal bovine serum, 0.1 U/L penicillin, and 100 g/L streptomycin sulfate. Killing was assessed using MTT to measure cell viability. The percent of specific cytotoxicity was calculated as follows:

#### Phenotyping

Populations of total T cells (CD3^+^CD19^-^), T cytotoxic cells (Tc; CD3^+^CD8^+^), T helper cells (Th; CD3^+^CD4^+^), B cells (CD3^-^CD19^+^), and natural killer cells (NK; CD3^-^/CD16^+^56^+^) were quantitated by dual color flow cytometry as previously described [10,11]. Cells were labeled with monoclonal antibodies conjugated to fluorescein isothiocyanate (FITC) or phycoerythrin (PE): anti-CD3 was conjugated to FITC, and anti-CD8, anti-CD4 and anti-CD19 were conjugated to PE (Caltag Laboratories, Burlingame, CA). In addition, the distribution of the intercellular adhesion molecule ICAM-1 (CD54^+^, BD Biosciences), and the leukocyte function antigens LFA-1 (CD11a^+^, BD Biosciences) and LFA-3 (CD58^+^, BD Biosciences) were measured. A lymphocyte analysis gate and the antibodies CD45-FITC and CD14-PE (Caltag Laboratories, Burlingame, CA) were used to help distinguish the lymphocytes from other blood cell types. A total of 2000 gated events were acquired for each sample and analyzed by flow cytometry (FACScan, BD Biosciences, San Jose, CA) using the Cell Quest program (version 3.3).

#### Tuberculin delayed-type hypersensitivity

Delayed-type hypersensitivity (DTH) response to an intracutaneous injection of tuberculin (Mono-Vacc Test O.T., Pasteur Merieux Connaught, France) was assessed on wk 8. A physician administered the injections and also measured skin thickness and induration at 0, 24, 48 and 72 h after challenge.

#### Cytokine production

Plasma samples were analyzed using commercially available ELISA kits for IL-2 (BD OptEIA™ Set Human IL-2, BD Biosciences, San Diego, CA), TNFα (BD OptEIA™ Set Human TNF), and IFN-γ (BD OptEIA™ Set Human IFN-γ), as well as IL-1β (Amersham Pharmacia Biotech Inc., Piscataway, NJ) and IL-6 (Amersham Pharmacia Biotech Inc.).

#### C-Reactive protein

C-Reactive protein (CRP), a well-established marker of inflammatory status, was measured in plasma with a commercially available ELISA (Alpha Diagnostic, San Antonio, TX).

#### Oxidative damage to DNA

Oxidative DNA damage was assessed by measuring plasma 8-hydroxy-2'-deoxyguanosine (8-OHdG) using competitive ELISA (BIOXYTECH^® ^8-OHdG-EIA Kit, OxisResearch, Portland, OR).

#### Lipid-peroxidation

Plasma concentrations of 8-epi-prostaglandin F2α (8-isoprostane) were measured by a commercially available competitive ELISA (8-Isoprostane EIA kit, Cayman Chemical Company, Ann Arbor, MI).

### Statistical analysis

Data were analyzed by repeated measures ANOVA using the General Linear Model of SAS [12]. Differences among treatment means were compared by a protected LSD test and considered different at *P < 0.05*.

## Results

### Plasma astaxanthin

**Figure 1:**
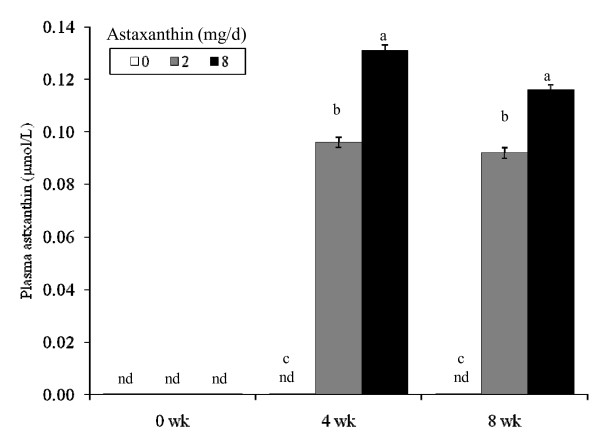
**Concentrations of plasma astaxanthin in human subjects fed 0, 2 or 8 mg astaxanthin daily for 8 wk**. ^a, b ^Different letters represent significant treatment differences (*P *< 0.05) as analyzed by protected LSD test. Values are means; variation is expressed as a representative overall standard error.

Astaxanthin was not detectable in the plasma of any subjects at wk 0 or in the conrol group at wk 4 or 8. However, concentrations of astaxanthin in plasma increased to maximal concentrations by wk 4 in a dose-dependent manner (Figure 1). Dietary recall showed no treatment difference in daily dietary intake (Table 1).

**Table 1:** Composition of averaged 3-day dietary recall within subject treatment groups (n = 14/diet treatment group) prior to daily supplementation with 0, 2 or 8 mg astaxanthin.

	Astaxanthin		
	
	0 mg	2 mg	8 mg
Total Kcal (Kcal/d)	1777	1618	1736
Protein (g/d)	64	59	68
Fat (g/d)	54	49	52
CHO (g/d)	258	238	254
			
α-Carotene (μg/d)	457	527	567
β-Carotene (μg/d)	1813	1664	1773
β-Cryptoxanthin (μg/d)	61	81	38
Lutein & Zeaxanthin (μg/d)	610	490	338
Lycopene (μg/d)	2067	2327	1133

There were no differences between groups, as determined by T test.

Values are means of 3-day dietary recalls.

### Lymphoproliferation

**Figure 2:**
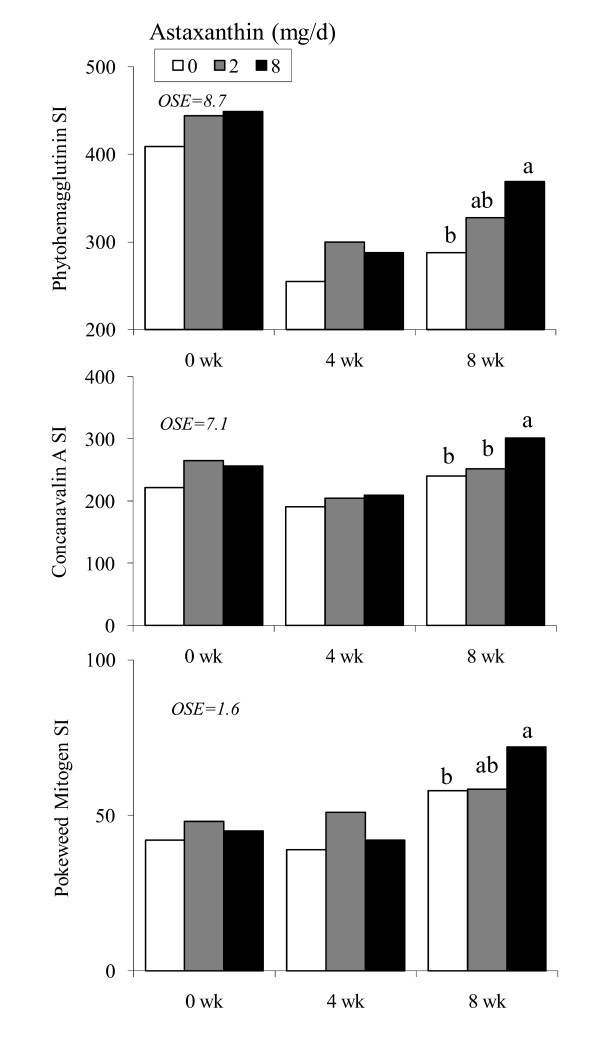
**Lymphocyte proliferation induced with phytohemagglutinin, concanavalin A and pokeweed mitogen in human subjects fed 0, 2 or 8 mg astaxanthin daily for 8 wk**. Responses to high concentrations of mitogens (10 mg phytohemmaglutinin/L, 10 mg concanavalin A/L and 5 mg pokeweed mitogen/L final concentration) are shown. ^a, b ^Different letters represent significant treatment differences (*P *< 0.05) as analyzed by protected LSD test. Values are means ± SEM.

Proliferation of peripheral blood mononuclear cells was consistently higher (*P *< 0.05) when stimulated with both T cell-dependent (phytohemmaglutinin, concanavalin A) and B cell-dependent (pokeweed mitogen) mitogens in 8Asta on wk 8 (Figure 2). Both concentrations of each mitogen showed similar trends whether mitogens were low or high concentration. No differences in response were observed in 2Asta.

### NK Cytotoxicity

Higher (*P *< 0.05) NK cell cytotoxic activity (effector:target cell ratio of 10:1) was seen in 8Asta but not in 2Asta by wk 8 (Table 2).

**Table 2:** Immune cell response following daily supplementation with 0, 2 or 8 mg astaxanthin after 0, 4 and 8 wk.

Treatment	Wk 0	Wk 4	Wk 8
Total T cells (%)			
0 mg	65.9 ± 2.1	64.4 ± 1.9^b^	70.6 ± 1.5^b^
2 mg	69.5 ± 1.4	69.6 ± 1.5^a^	75.7 ± 1.6^a^
8 mg	67.2 ± 2.2	69.6 ± 1.8^a^	74.3 ± 1.8^ab^
Total T_h _cells (%)			
0 mg	35.3 ± 1.5	36.3 ± 1.7	39.6 ± 1.4
2 mg	35.3 ± 1.8	36.0 ± 1.9	39.7 ± 2.0
8 mg	35.2 ± 1.5	34.3 ± 1.4	38.4 ± 1.2
Total T_c _cells (%)			
0 mg	26.7 ± 1.4	26.1 ± 1.2	28.4 ± 1.4
2 mg	28.2 ± 1.9	27.6 ± 1.9	28.7 ± 1.6
8 mg	28.4 ± 1.6	28.3 ± 1.6	28.8 ± 2.1
T_h_:T_c _ratio cells			
0 mg	1.35 ± 0.07	1.43 ± 0.09	1.48 ± 0.10
2 mg	1.31 ± 0.15	1.35 ± 0.16	1.46 ± 0.15
8 mg	1.31 ± 0.13	1.29 ± 0.13	1.37 ± 0.14
Total B cells (%)			
0 mg	11.4 ± 0.9	11.1 ± 0.5	10.7 ± 0.5^b^
2 mg	13.1 ± 1.1	12.7 ± 1.2	13.1 ± 0.5^a^
8 mg	13.3 ± 1.4	11.9 ± 0.7	11.1 ± 0.3^ab^
Total NK cells (%)			
0 mg	13.7 ± 0.9	12.6 ± 1.2	10.6 ± 0.8
2 mg	12.5 ± 1.0	13.1 ± 1.9	12.6 ± 1.5
8 mg	13.4 ± 1.1	15.6 ± 1.4	13.1 ± 1.4
NK cytotoxic activity(% lysis)			
0 mg	51.5 ± 4.4	51.0 ± 2.5	57.8 ± 2.7^b^
2 mg	58.2 ± 3.8	54.4 ± 2.9	57.1 ± 2.6^b^
8 mg	56.1 ± 3.1	54.3 ± 1.9	67.9 ± 3.0^a^

^a, b ^Different letters represent significant treatment differences (*P *< 0.05) as analyzed by ANOVA. Values are means ± SEM.

### Phenotyping

The population of CD3+ total T cells was higher (*P *< 0.05) in 2Asta or 8Asta compared to Con on both wk 4 and 8 (Table 2). The percent B cell population was higher (*P *< 0.05) only in 2Asta on wk 8 (Table 2). On the other hand, dietary astaxanthin did not significantly influence the population of Th, Tc or NK cells or the ratio of Th:Tc cells (Table 2).

On wk 8, the frequency of cells expressing CD11a+ LFA-1 marker was higher in 2Asta (42%) but not those given 8Asta (30.6%) compared to Con (32%). Supplemental astaxanthin did not have a significant effect on the expression of the cell surface adhesion molecules ICAM-1 (CD54) and LFA-3 (CD58) (data not shown).

### Tuberculin DTH test

**Figure 3:**
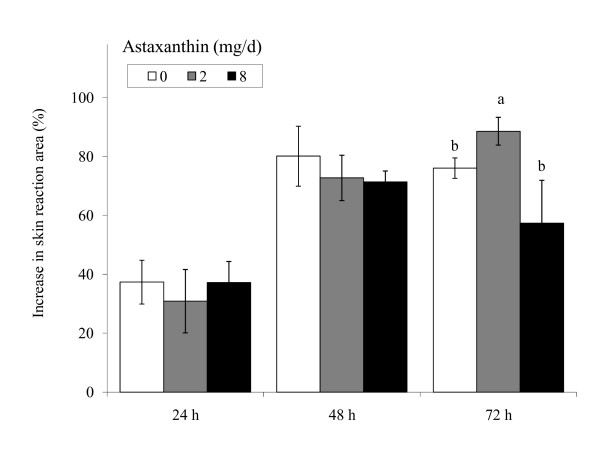
**Delayed-type hypersensitivity tuberculin test in human subjects fed 0, 2 or 8 mg astaxanthin daily for 8 wk**. ^a, b ^Different letters represent significant treatment differences (*P *< 0.05) as analyzed by protected LSD test. Values are means ± overall standard error.

DTH response was maximal at 48 to 72 h post-challenge (Figure 3). Subjects fed 2Asta had higher (*P *< 0.05) DTH response than Con, whereas 8Asta did not show a similar DTH response.

### Cytokines

No differences in TNF-α and IL-2 levels were seen in any treatments (Table 3). By wk 8, IFN-γ concentrations were higher in 8Asta (*P *< 0.05) (Table 3); IFN-γ in 2Asta and Con did not change throughout the study. Treatment 8Asta also showed an increase (*P *< 0.05) in IL-6 on wk 8.

**Table 3:** Cytokine response following daily supplementation with 0, 2 or 8 mg astaxanthin after 0, 4 and 8 wk.

Treatment	Wk 0	Wk 4	Wk 8
TNF-α (pg/mL); *Overall SE = 0.43*			
0 mg	1.14	1.63	1.43
2 mg	0.80	1.13	1.44
8 mg	1.51	2.15	2.60
IFN-γ (pg/mL); *Overall SE = 0.34*			
0 mg	5.85	4.47	4.68^b^
2 mg	4.87	4.26	5.00^b^
8 mg	4.67	6.23	9.55^a^
IL-6 (pg/mL); *Overall SE = 2.9*			
0 mg	10.5	12.7	13.6^b^
2 mg	10.0	11.5	8.7^b^
8 mg	11.5	12.4	25.2^a^
IL-2 (pg/mL); *Overall SE = 0.10*			
0 mg	8.61	8.04	7.74
2 mg	5.09	4.67	4.46
8 mg	4.91	4.01	3.90

^a, b ^Different letters represent significant treatment differences (*P *< 0.05) as analyzed by ANOVA. Values are means; variation is expressed as a representative overall standard error.

### C-Reactive protein

**Figure 4:**
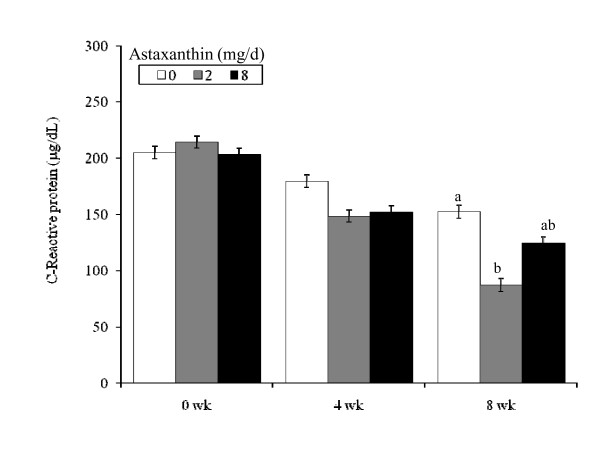
**Plasma concentrations of plasma C-reactive protein in human subjects fed 0, 2 or 8 mg astaxanthin daily for 8 wk**. ^a, b ^Different letters represent significant treatment differences (*P *< 0.05) as analyzed by protected LSD test. Values are means ± overall standard error.

The concentration of plasma C-reactive proteins was lower (*P *< 0.05) in 2Asta on wk 8 compared to the Con (Figure 4). However, higher dietary astaxanthin amounts did not influence the concentration of this acute phase protein.

### DNA Damage

**Figure 5:**
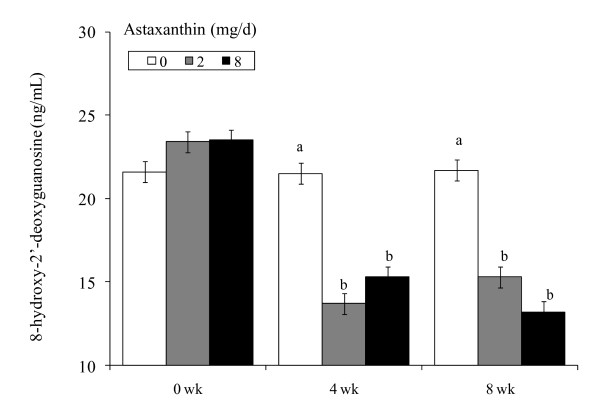
**Concentrations of plasma 8-hydroxy-2'-deoxyguanosine in human subjects fed 0, 2 or 8 mg astaxanthin daily for 8 wk**. ^a, b ^Different letters represent significant treatment differences (*P *< 0.05) as analyzed by protected LSD test. Values are means ± overall standard error (OSE = Root MSE÷square root of n+1).

Concentrations of 8-OHdG were dramatically lower (*P *< 0.01) as early as wk 4 in 2Asta and 8Asta (Figure 5). DNA damage observed with 2Asta was not further decreased in the group fed higher dietary astaxanthin (8Asta).

### Lipid Peroxidation

Dietary astaxanthin did not significantly influence concentrations of plasma 8-isoprostane at all periods studied. The overall mean concentration of 8-isoprostane was 30.8 ± 0.4 pg/mL across all treatments.

## Discussion

While the biological action of astaxanthin has been reported in both *in vitro *and *in vivo *studies, these have mainly used rodents and *in vitro *models. This is the first comprehensive study to examine the action of dietary astaxanthin in regulating immune response, oxidative damage and inflammation in humans. Dietary astaxanthin enhanced both cell-mediated and humoral immune responses in young healthy feamles. The immune markers significantly enhanced by feeding astaxanthin included T cell and B cell mitogen-induced lymphocyte proliferation, NK cell cytotoxic activity, IFN-γ and IL-6 production, and LFA-1 expression. Enhancement of these *ex vivo *immune markers corresponded with increased number of circulating total T and B cells. In addition, subjects given astaxanthin also showed an enhanced tuberculin DTH response, a reliable clinical test to assess *in vivo *T cell function. All of these immune responses were generally observed after 8 wk of supplementation following a cutaneous tuberculin injection.

Modulatory actions of astaxanthin on immune response have been demonstrated in both *in vitro *and *in vivo *studies. We previously reported higher mitogen-induced splenocyte proliferation in mice [4], dogs [13] and cats [14] fed astaxanthin. Astaxanthin stimulated cell proliferation of murine splenocytes and thymocytes in vitro [15]. Others have shown that astaxanthin increased cytotoxic T lymphocyte activity in mice [16] and inhibited stress-induced suppression of NK cell activity [17]. In this study, astaxanthin heightened NK cell cytotoxic activity. Natural killer cells serve in an immuno-surveillance capacity against tumors and virus-infected cells; therefore, astaxanthin may play a role in cancer etiology. Patients with Chediak-Higashi syndrome, a disorder associated with defective NK cell function, are indeed more susceptible to tumor formation.

Flow cytometry data showed higher subpopulations of total T and B cells. Activated T cells and NK cells produce IFN-γ, which is involved in immune-regulation, B cell differentiation, and antiviral activity. IFN-γ production was higher in subjects supplemented with astaxanthin, similar to the response in mice given astaxanthin [16]. Splenocytes of tumor-bearing mice fed lutein also had higher IFN-γ expression, and these changes paralleled the inhibitory action of lutein against tumor growth [18]. Astaxanthin decreased bacterial load and gastric inflammation in mice infected with *Helicobacter pylori *by shifting the T-lymphocyte response from a Th1 response dominated by IFN-γ to a Th1/Th2 response dominated by IFN-γ and IL-4 [5]. Modulation of the humoral immune response also occurs; astaxanthin increased antibody production in mouse splenocytes [19], partially restored humoral immune response in old mice [6], enhanced immunoglobulin production in response to T-dependent stimuli in human blood cells [20] and induced production of polyclonal antibodies G and M in murine spleen cells [15]. The present study suggests that the higher antibody production may be due to an increase in B cell number.

The skin tuberculin test is a reliable clinical test to assess *in vivo *T cell function. This study shows that subjects given astaxanthin had a heightened DTH response, which is also seen in dogs [13] and cats fed astaxanthin [14], β-carotene [10,14] and lutein [21,22]. Leukocyte function antigens (LFA) mediate intercellular adhesion between leukocytes and other cells in an antigen non-specific fashion. LFA-1 is a β_2_-integrin expressed on leukocytes involved in the migration of lymphocytes, monocytes and neutrophils. LFA-1 binds to ICAM-1 and ICAM-2 expressed on vascular endothelium, and controls lymphocyte migration into inflammatory sites. Endothelial expression of ICAM-1 is inducible while ICAM-2 is constitutive. Therefore, the heightened DTH response in this study is likely due to an increased expression of LFA-1 but not ICAM.

Dietary astaxanthin dramatically decreased one DNA damage biomarker (plasma 8-OHdG), and this protective effect was observed by wk 4 of feeding. Maximal response was observed with the lower 2 mg astaxanthin dose. In addition, subjects fed 2 mg astaxanthin also showed lower plasma C-reactive protein concentrations, demonstrating the anti-inflammatory action of astaxanthin in humans. Immune cells are particularly sensitive to membrane damage by free radicals. Reactive oxygen species (ROS) are produced via the mitochondria electron transport system during ATP production, xanthine oxidase and phagocytes [23,24]. In fact, cumulative oxidative damage to the mitochondria is considered the main culprit of cell senescence which in turn is responsible for aging and the development of age-related chronic diseases [25]. The ROS can induce redox-sensitive transcription factors such as NFkB and AP-1, which regulate genes controlling production of chemokines, inflammatory cytokines, and adhesion molecules which stimulate phagocytic infiltration [26]. Conversely, astaxanthin, acting as a potent antioxidant, can inhibit ROS-induced production of these transcription factors, thereby decreasing inflammation. Indeed, astaxanthin attenuated exercise-induced neutrophil infiltration and subsequent delayed-onset damage to the gastronemius and heart muscle in mice [26]. Astaxanthin is reported to be approximately 100 fold more protective than lutein and β-carotene against UVA-induced oxidative stress *in vitro *[8].

Reactive nitrogen species also play an important role in inflammation. As with ROS, astaxanthin has been reported to decrease the production of nitric oxide (NO) and iNOS activity in a mouse macrophage cell line, resulting in the inhibition of COX which down-regulates the production of PGE_2 _and TNF-α [27]. TNF-α is a pleiotropic cytokine produced by activated macrophages and monocytes, and has nonspecific resistance against various infectious agents. Similarly, Lee *et al*. [28] reported that astaxanthin suppressed serum NO, TNFα and IL-1β in mice injected with lipopolysaccharide. The TNF-α and IL-1 cascade activates p38 MAPK, thus promoting proinflammatory gene expression and cytokine production. Therefore, in this study, astaxanthin exerted its anti-inflammatory action by inhibiting reactive oxygen and nitrogen species.

Why dietary astaxanthin did not reduce lipid peroxidation is unclear. Astaxanthin has been shown to be one of the most effective antioxidants against lipid peroxidation and oxidative stress in *in vitro *and *in vivo *systems [3,29]. Humans given 1.8 to 21.6 mg astaxanthin daily for 14 d increased the lag time for LDL oxidation [30]. Astaxanthin is as effective as α-tocopherol in inhibiting radical-initiated lipid peroxidation in rat liver microsomes [31], and is 100 times more active than α-tocopherol in protecting the rat mitochondria against Fe^2+^-catalyzed lipid peroxidation *in vivo *and *in vitro *[3]. The potent antioxidant activity of astaxanthin is likely due to the presence of a keto- and a hydroxyl group on each end of its molecule. This structural property effectively rigidifies cell membranes, thereby limiting the penetration of lipoperoxidation promoters across the lipid bilayer [32]. The isoprostane methodology used in this study lacks sensitivity and accuracy; this may account for the lack of a significant effect seen in this study.

Taken together, the immunomodulatory, antioxidative and anti-inflammatory activity of astaxanthin will likely influence the etiology of cancer and inflammatory diseases. Astaxanthin was more active than β-carotene, lutein and canthaxanthin in inhibiting mammary tumor growth in mice [7]. Others have reported that astaxanthin protected against carcinogenesis of the urinary bladder [33], decreased cancerous growth of the mouth [34], and decreased the number and size of liver preneoplastic foci [35] in rodents. Astaxanthin, as an algal extract, protected UVA-induced DNA damage in human skin fibroblasts (IBR-3), melanocytes (HEMAc) and intestinal CaCo2 cells [36]. In addition, astaxanthin ameliorated other oxidative stress-induced inflammatory diseases such as diabetic nephropathy in diabetic mice [37], lipopolysaccharide-induced uveitis in rats [27], and exercise-induced skeletal and cardiac muscle damage in mice [26].

The polar ends if the astaxanthin structure allows it span biological membranes; this transmembrane alignment allows astaxanthin to preserve the membrane structure [38], decrease membrane fluidity [39], and function as an antioxidant [40]. These and other mechanisms may explain the antioxidative, anti-inflammatory and immune-modulatory action of astaxanthin.

In this study, plasma concentrations of astaxanthin in subjects given 2 or 8 mg astaxanthin daily for 4 wk increased to 0.09 to 0.13 μmol/L, with no further increase observed at 8 wk. These plasma concentrations are lower than that reported by Osterlie *et al*. [41] who showed maximal concentrations of 1.3 mg/L (2.28 μmol/L) in subjects administered a single oral dose of 100 mg astaxanthin. The difference in plasma concentrations in the two human studies is expected, due to differences in the dose and length of astaxanthin administration; however, the two studies also used different sources of astaxanthin and different subject gender and age. Most of the astaxanthin was found in the VLDL chylomicra, with lesser amounts in the LDL and HDL [41]. While the stereoisomer form of astaxanthin was not identified in this study, Osterlie *et al*. [41] reported a preferential uptake of the Z-isomers as compared to the all-E-astaxanthin. Astaxanthin used in the present study is from *Haematococcus pluvialis *and exists primarily in an esterified 3S, 3'S stereoisomer while synthetic astaxanthin [41] is primarily the 3R, 3'S form. The amount of supplemental astaxanthin used in this study is achievable through diet means. For instance, the astaxanthin content of salmon flesh ranges from 3 to 37 mg/kg [42,43]; therefore, a 200-g serving of salmon provides approximately 1 to 7 mg astaxanthin. Wild salmon contains the 3S, 3S' form of astaxanthin almost exclusively. The 3R, 3R' form is found rarely in nature but does exist in some crustaceans such as in Krill. In healthy humans, 6 mg astaxanthin from *H. pluvialis *algal extract can be safely consumed [44].

Overall, this study shows that dietary astaxanthin enhanced immune response, and decreased a DNA oxidative damage biomarker and inflammation in young healthy females. It is the initial scope of the study to focus on a narrow population with regards to age, gender and race; however, antioxidants generally show greater physiologic modulation under excess amounts of oxidative stress, in immuno-compromised individuals, and with longer feeding periods. These likely explain the lack of efficacy in certain response measures studied. Future studies with astaxanthin administration will include these parameters. However, our present study suggests astaxanthin to be a bioactive natural carotenoid that may be important to human health.

## Competing interests

The authors declare that they have no competing interests.

## Authors' contributions

JSP and BPC designed research, analyzed data, and wrote the paper; JSP, BPC, JHC, and YKKconducted research; LLL provided essential materials; BPC had primary responsibility for final content. All authors read and approved the final manuscript.
